# Optimal waist circumference threshold for diagnosing metabolic syndrome in African people living with HIV infection

**DOI:** 10.1371/journal.pone.0183029

**Published:** 2017-09-08

**Authors:** Kim A. Nguyen, Nasheeta Peer, Anniza de Villiers, Barbara Mukasa, Tandi E. Matsha, Edward J. Mills, Andre P. Kengne

**Affiliations:** 1 Non-Communicable Diseases Research Unit, South African Medical Research Council, Cape Town, South Africa; 2 Department of Medicine, University of Cape Town, Cape Town, South Africa; 3 United Nations Population Fund (UNFPA), Mildmay, Uganda; 4 Department of Biomedical Sciences, Faculty of Health and Wellness Science, Cape Peninsula University of Technology, Cape Town, South Africa; 5 Global Evaluation Science, Vancouver, Canada; Temple University School of Medicine, UNITED STATES

## Abstract

**Background:**

The applicability of the internationally advocated cut-off points of waist circumference (WC) derived from Caucasians to diagnose metabolic syndrome (MS) in HIV-infected Africans is unknown. This study aimed to determine the optimal WC cutoffs for MS diagnosis in HIV-infected people receiving care at public healthcare facilities in the Western Cape Province in South Africa.

**Methods:**

Data from 748 randomly selected participants (591 women), with a median age of 38 years, were analysed. The Youden’s index and the top-left-point approaches were used to determine the optimal cutoffs of WC for predicting ≥2 non-adipose MS components.

**Results:**

The two approaches generated the same WC cut-off point in women, 92 cm (sensitivity 64%, specificity 64%) but different cut-off points in men: 87 cm (sensitivity 48%, specificity 85%) based on the Younden’s index and 83 cm (sensitivity 59%, specificity 74%) by the top-left-point method. The advocated thresholds of 94 cm in men had low sensitivity (30%) but high specificity (92%) whereas 80 cm in women showed low specificity (32%) but high sensitivity (85%) for diagnosing MS in this sample. Most African-specific cut-off points performed well, with 90 cm providing acceptable performance in both men (sensitivity 43%, specificity 88%) and women (sensitivity 66%, specificity 59%).

**Conclusions:**

This study underlines the sub-optimal performance of internationally recommended WC thresholds for MS diagnosis in HIV-infected Africans, and supports the need to revisit the guidelines on WC criterion in African population across the board. A single threshold of 90 cm for both genders would be a practical suggestion.

## Introduction

Metabolic syndrome (MS) represents the constellation of cardio-metabolic risk factors that includes abnormal fat distribution, dyslipidaemia, hyperglycaemia, and hypertension. Since it was first described by Reaven in 1988, several international organisations, and expert groups have proposed diagnostic criteria for the MS based on variable combinations of cardio-metabolic risk factors and at times, at variable thresholds [1]. Popular diagnostic criteria include those of the World Health Organization (WHO) in 1998, the European Group for the Study of Insulin Resistance (EGIR) in 1999, the Adult Treatment Panel III (ATPIII) (2001, 2004, 2005), the International Diabetes Federation (IDF) in 2005, and most recently in 2009 the harmonized Joint Interim Statement (JIS) [1].

Body fat distribution is an important criterion in the MS definition, but variably captured across diagnostic criteria. Body mass index (BMI) and waist-to-hip ratio (WHR) were indicators of body fat used in the WHO criteria, [2] while waist circumference (WC) has been recommended as a surrogate for abnormal fat distribution in more recent MS criteria [1]. Using WC over BMI and other markers of adiposity emphasised the important role of central obesity in the development of cardiovascular diseases (CVD) and diabetes mellitus, which are major consequences of the MS. Furthermore, WC is the simplest clinical measure of fat distribution [3]. However, selecting the most appropriate WC thresholds to diagnose MS has appeared to be complex due to its likely dependence on gender, and ethnicity [4]. Although the IDF and JIS criteria have attempted to incorporate ethnic-specific cut-off points for WC in the MS definition, these criteria advocate using Europid thresholds (men ≥94cm, women ≥80cm) in African people in the absence of specific thresholds for African populations [3]. However, emerging evidence from few cross-sectional studies across Africa are not in support of uncritical application of Europid WC thresholds in African populations [5–9]. These studies have been consistent in suggesting that their application will likely results in over-diagnosis of women and under-diagnosis of men with abdominal obesity and accordingly, the MS.

In the era of highly active antiretroviral therapy (ART), people with HIV infection, the majority of whom are found in Africa, are increasingly living longer, with cardio-metabolic diseases becoming a new threat to their healthy survival. As many as 46% of HIV-infected people have hypertension, [10] and nearly one third of them have MS regardless of the diagnostic criteria [11]. Considering that HIV infection and related treatments are associated with modifications of the distribution of body fat and metabolic factors, it is essential to confirm the applicability of recommended WC thresholds for MS diagnosis in people with HIV infection, or derived news ones with improved diagnostic accuracy for MS, particularly in African people, in view of informing appropriate screening and management of the condition. Therefore, this study aimed to determine the optimal WC cut-off points for MS diagnosis in patients receiving HIV-care at public healthcare facilities in the Western Cape Province in South Africa.

## Material and methods

### Study population and sampling

The data for the present analysis are from a cross-sectional study conducted among HIV-infected men and women aged 18 years and older, receiving care at primary healthcare facilities in the Western Cape Province, South Africa. The study methods have been described in detail elsewhere [12]. In brief, the patients were selected from 17 public healthcare facilities including ten in Cape Town and seven in the surrounding rural municipalities, applying random sampling procedures. Patients were included in the study if they were not pregnant or breastfeeding, bedridden, undergoing treatment for cancer, nor on corticosteroid treatment, and were willing and able to give consent. The study was approved by the South African Medical Research Council Ethics Committee and conducted in accordance with the principles of the Declaration of Helsinki. The Health Research Office of the Western Cape Department of Health, and the selected healthcare facilities granted permission to conduct the survey.

### Data collection

The data were collected between March 2014 and February 2015 by a team of trained clinicians, nurses and fieldworkers, and captured on personal digital assistants (PDAs), using electronic case report forms with built-in checks for quality control. These were then encrypted at the point of collection and sent via mobile connections to a dedicated server where it was further checked, downloaded and stored for future use. A structured interviewer-administered questionnaire adapted from the World Health Organization’s (WHO) STEPwise approach to Surveillance (STEPS) tool was used to obtain socio-demographic and medical history. Duration of diagnosed HIV infection, CD4 counts and ART regimens were obtained from participants’ records.

#### Anthropometric and blood pressure measurements

Anthropometric measurements were taken using standardised techniques. Heights and weights were measured with the participants in light clothing and bare-footed. Waist and hip circumferences (HC) were measured to the nearest millimeter at the level of umbilicus and around the largest circumference of the buttocks, respectively. Blood pressure (BP) was measured on the right arm, using a digital BP monitor (Omron, M6 Comfort, Netherland) after the participant was seated in a resting position for at least five minutes; three measurements were taken three minutes apart, and the average of the 2^nd^ and 3^rd^ readings used in the analysis.

#### Biochemical analysis

Fasting blood samples were drawn via venepuncture. All lipid and glucose concentrations were measured with an autoanalyser, Beckman Coulter AU 500 spectrophotometer. Serum lipid triglycerides and high-density lipoprotein cholesterol (HDL-C) were analysed by enzymatic colorimetric method; plasma glucose was measured by hexokinase method.

#### Definitions

The individual components of the MS and their cut-off points were defined according to the JIS criteria: elevated WC: men≥94 cm, women≥80 cm; elevated triglycerides: ≥1.7 mmol/L; low HDL-C: men <1.03 mmol/L, women <1.3 mmol/L; elevated BP: ≥130/85 mmHg or on hypertensive medication; hyperglycaemia: fasting glucose≥5.6mmol/L or on glucose control agents. WHR was calculated as WC (cm) divided by HC (cm) and waist-to-height ratio (WHtR) as WC (cm) divided by height (cm).

### Statistical analysis

Data analyses, using R statistical software, version 3.0.3 (2014-03-06) (The R Foundation for Statistical Computing Platform, Vienna, Austria), were stratified by gender to account for gender related differences in body fat distribution. [1] Continuous variables are presented as means (± standard deviation, SD) or medians (25^th^-75^th^ percentiles), and categorical variables as frequencies and percentages. Mann-whitney U test and chi square test were used for men vs. women comparisons. The “pROC” package was used for receiver operating characteristic curves (ROC) analyses. The area under the curve (AUC) was used to assess and compare the ability of WC and other adiposity variables to detect the presence of two or more non-adipose components of the MS. The AUC values range between 0 and 1; an AUC value closer to 1 infers a better ability of the predictor/test to discriminate individuals with and without the defined condition or disease, whereas an AUC value of 0.5 indicates no discriminative power of the predictor/test of interest [13]. In the ROC curve, pairs of the false positive rate (1-specificity) and the true positive rate (sensitivity) for every individual cut-off point are plotted. The shape of the ROC curve indicates how high the discriminatory power of the test is; perfect discrimination has an ROC curve that passes through the upper left-hand corner (100% sensitivity, 100% specificity). Thus, the closer the ROC curve is located to the upper left-hand corner and the larger the AUC, the higher the overall accuracy of the test. The optimal WC was determined by applying both the Youden’s index approach and the closest-top-left-hand point approach [14]. The Youden’s index is calculated by sensitivity + specificity– 1, and ranges from 0 to 1, with values approaching 1 indicating a better performant test and vice versa. Maximizing this index (J-point) allows finding an optimal level independently from the outcome prevalence.

The diagnostic performance of the cut-off points derived in this study were assessed alongside the internationally advocated thresholds and those from other South African and African studies at large, by computing a number of diagnostic performance measures including the ***sensitivity*** *(se)* which is the probability of a positive test result in a person with the disease/target condition; the ***specificity*** *(sp)*, the probability of a negative test result in a person without the disease/target condition; the ***positive predictive value*** (PPV), the probability of having the disease in a person with a positive test; the ***negative predictive value*** (NPV), the probability of no disease in a person with negative test; the ***likelihood ratio for positive test result*** (LR+), representing how much more likely the positive test result will occur in persons with the disease/condition compared to those without the disease and ***likelihood ratio for negative test results*** (LR-), how less likely the negative test result is to occur in persons with the disease than in those without the disease [15]. Additionally, global measures such as the ***Youden’s index*, *diagnostic odds ratio*** (DOR, the ratio LR+/LR-); ***diagnostic accuracy***, expressing percentage of correctly classified subjects among all subjects; and ***number needed to diagnose*** (NND, = 1/Youden’s index) were also computed. Performance measures’ calculation used the “epiR” package of R.

## Results

### Characteristics of the participants

**Fig 1:**
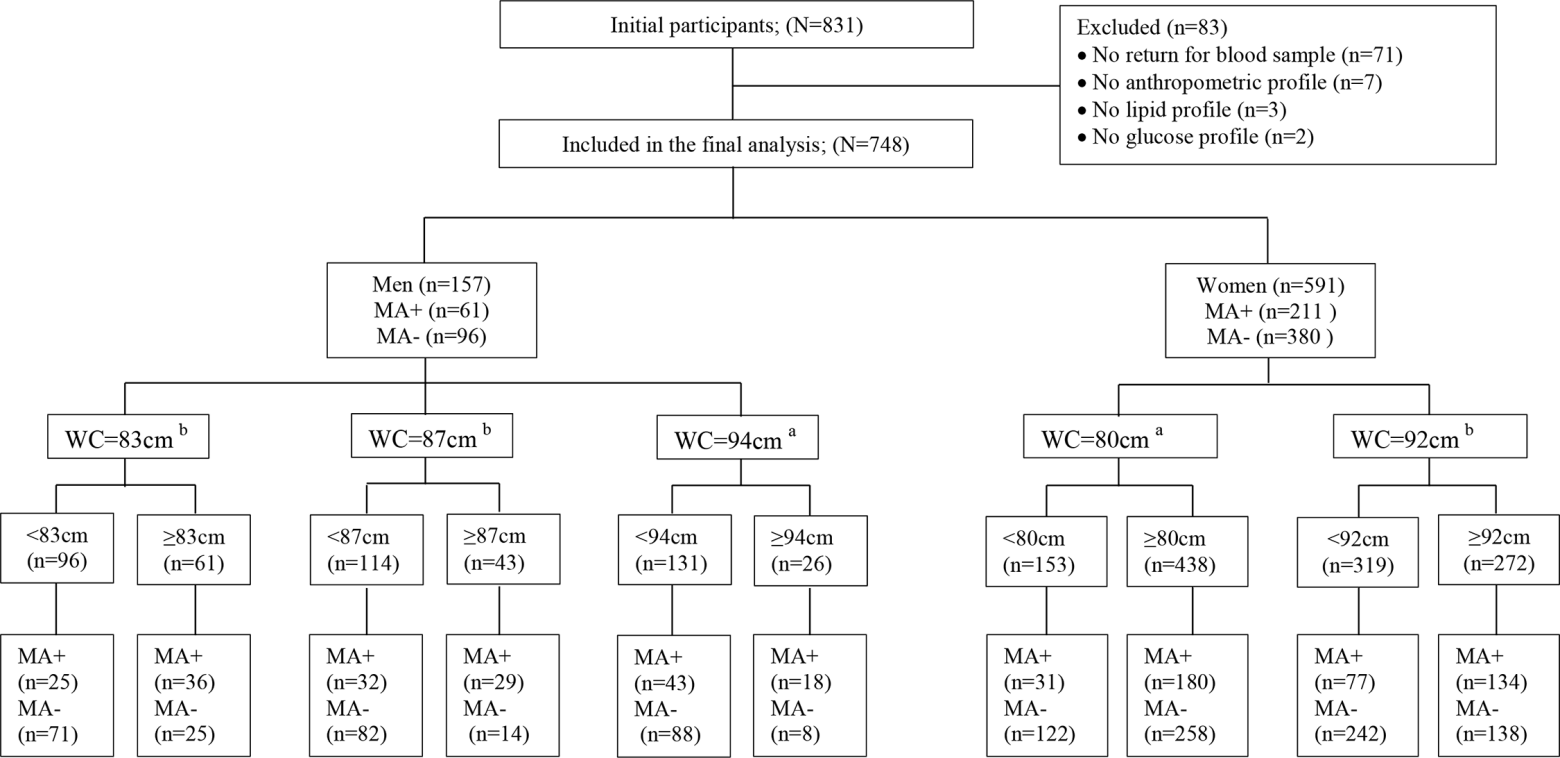
Standard of Reporting for Diagnostic Accuracy Studies (STARD) diagram to report flow of participants through the study. WC, waist circumference; MA, metabolic abnormalities, ≥2 non-adipose components: BP≥130/85mmHg, triglycerides≥1.7mmol/L, high density lipoprotein cholesterol<1.03mmol/l in men and <1.3mmol/l in women, fasting glucose≥5.6mmol/l; ^a^ WC cutoffs recommended by the JIS criteria; ^b^WC cutoffs derived in the present study.

The Standard of Reporting for Diagnostic Accuracy Studies (STARD) diagram for the flow of participants in the study is shown in Fig 1. Data for 748 participants, comprising 157 men (21%) and 591 women (79%), were analysed. Table 1 shows the basic characteristics of the study participants. Men, with a median age of 41 years, were significantly older than women whose median age was 37 years (p<0.001). However, women compared with men had a longer duration of diagnosed HIV infection (5 years vs. 4 years, p<0.001) and higher CD4 count levels (410 cells/mm^3^ vs. 272 cells/mm^3^, p = 0.002). Most (93%) of the study participants were on ART with no difference by gender (p = 0.296). Compared to men, women had higher BMI, larger WC, HC and WHtR but lower WHR, Table 1.

**Table 1:** HIV-related characteristics, and median anthropometric and biochemical parameters in the study sample.

Variables	Overall (N = 748)	Men (n = 157)	Women (n = 591)	p-value
Age (years)	38 (32–44)	41 (35–47)	37 (31–43)	<0.001
*Anthropometry*				
WC (cm)	88 (78–98)	78.9 (74–88)	90 (80–101)	<0.001
Hip (cm)	102 (93–112)	92 (88–97)	105 (97–115)	<0.001
WHR	0.86 (0.80–0.91)	0.87 (0.84–0.93)	0.85 (0.80–0.90)	<0.001
WHtR	0.55 (0.48–0.61)	0.47 (0.44–0.52)	0.57 (0.50–0.63)	<0.001
BMI (kg/m^2^)	26.3 (22.1–32)	21.4 (19.8–22.4)	28.3 (23.8–28.9)	<0.001
*Blood pressure*				
Systolic (mmHg)	117 (107–130)	123.5 (114.5–140)	115 (105.8–127)	<0.001
Diastolic (mmHg)	82 (75–91)	83 (76–94)	81.5 (74.8–89.8)	0.129
*Glucose*				
FPG (mmol/L)	5 (4.6–5.4)	5.1 (4.8–5.5)	4.9 (4.6–5.4)	0.010
*Lipids*				
Triglycerides (mmol/L)	1 (0.7–1.3)	1.12 (0.75–1.27)	0.97 (0.74–1.28)	0.023
HDL-C (mmol/L)	1.3 (1–1.5)	1.2 (1.0–1.5)	1.29 (1.08–1.52)	0.010
*Prevalence of MS components*, % (95%CI), JIS criteria				
Raised WC ^a^	62.0 (58.6–65.5)	16.6 (10.8–22.4)	74.1 (70.6–77.6)	<0.001
Hypertension^b^	46.3 (42.7–49.8)	46.5 (38.7–54.3)	46.2 (42.2–50.2)	0.946
Decreased HDL-C^c^	46.3 (42.7–49.8)	31.9 (24.6–39.1)	50.1 (46.1–54.1)	<0.001
Elevated Triglycerides^d^	13.6 (11.2–16.1)	22.9 (16.4–29.5)	11.2 (8.6–13.7)	<0.001
Hyperglycemia^e^	20.7 (17.8–23.6)	22.9 (16.4–29.5)	20.1 (16.9–23.4)	0.443
≥2 MS components	36.4 (32.9–39.8)	38.9 (31.2–46.5)	35.7 (31.8–39.6)	0.466
*HIV-related factors*				
HIV duration (years)	5 (2–9)	4 (2–7)	5 (2.5–9)	<0.001
CD4 count (cells/mm^3^)	392(240–604)	272 (193–448)	410 (253–627)	0.001
ART-related factors, n (%)	N = 699	N = 149	N = 550	0.296
ART-naive	46/699 (6.6)	7/149 (4.7)	39/550 (7.1)	
ART-treated	653/699 (93.4)	142/149 (95.3)	511/550 (92.9)	

ART, antiretroviral therapy; BMI, body mass index; FPG, fasting plasma glucose; HDL-C, high-density lipoprotein cholesterol; HIV, human immunodeficiency virus; LDL-C, low-density lipoprotein cholesterol; MS, metabolic syndrome, WHtR, waist-to-height ratio; WHR, waist-to-hip ratio.

^a^WC, waist circumference ≥94cm in men & ≥80cm in women

^b^Blood pressure≥130/85 mmHg or on hypertensive medication

^c^HDL-C, high density lipoprotein-cholesterol<1.03mmol/L in men & <1.3 mmol/L in women

^d^triglycerides ≥1.7mmol/L

^e^FPG ≥5.6mmol/L or on antidiabetic medication

The prevalence of the non-adipose components of the MS according to JIS criteria is presented in Table 1. Raised BP and low HDL-C levels were the most prevalent (46.3%), followed by hyperglycaemia (20.7%) and elevated triglycerides (13.6%). Raised BP and hyperglycaemia were similar in men and women (both p≥0.443) while low HDL-C was more common in the latter (women: 50.1% vs. men: 31.9%, p<0.001) and elevated triglycerides more frequent in the former (men: 22.9% vs. women: 11.2%, p<0.001). Notably, while the prevalence of raised WC was significantly different in women and men according to the JIS criteria (74% vs. 16.6%, p<0.001), the prevalence of ≥2 non-adipose components of the MS by the same criteria was similar (35.7% vs. 38.9%; p = 0.466).

### Discriminatory power of adiposity markers to detect two or more non-adipose MS components

Table 2 shows the discriminatory powers of different obesity indices in detecting ≥2non-adipose components of the MS. The highest point estimates of the AUC among the indices was recorded for WHR in men [0.723 (95%CI: 0.640–0.807)] and WHtR in women [0.657 (0.612–0.703)]. However, in both cases, the difference compared with WC was not significant (both p≥0.087). The AUC for WC was 0.659 (0.565–0.754) in men and 0.649 (0.603–0.695) in women. This was greater than the AUC for BMI found in women. HC showed the lowest predictive ability with AUCs of 0.590 (0.494–0.686) in men and 0.583 (0.535–0.631) in women with significant differences when compared with the other markers (all p<0.016), except with WHR in women (p = 0.107).

**Table 2:** Comparative abilities of adiposity markers to predict two or more metabolic syndrome components.

	AUC (95% CI)	vs. WC	vs. HC	vs. WHR	vs. WHtR	vs. BMI
***Overall***						
Waist circumference	0.646 (0.605–0.688)	-	<0.001	0.772	0.874	<0.001
Hip circumference	0.569 (0.526–0.612)	<0.001	-	0.006	<0.001	<0.001
Waist-to-hip ratio	0.652 (0.611–0.693)	0.772	0.006	-	0.816	0.106
Waist-to-height ratio	0.647 (0.606–0.688)	0.874	<0.001	0.816	-	<0.001
Body mass index	0.609 (0.567–0.651)	<0.001	<0.001	0.106	<0.001	-
***Men***						
Waist circumference	0.659 (0.565–0.754)	-	0.011	0.087	0.071	0.663
Hip circumference	0.590 (0.494–0.686)	0.011	-	0.016		0.008
Waist-to-hip ratio	0.723 (0.640–0.807)	0.087	0.016	-	0.398	0.230
Waist-to-height ratio	0.694 (0.604–0.783)	0.071	0.003	0.398	-	0.457
Body mass index	0.672 (0.583–0.762)	0.663	0.008	0.230	0.457	-
***Women***						
Waist circumference	0.649 (0.603–0.695)	-	<0.001	0.577	0.208	0.007
Hip circumference	0.583 (0.535–0.631)	<0.001	-	0.107	<0.001	0.001
Waist-to-hip ratio	0.637 (0.590–0.684)	0.577	0.107	-	0.355	0.513
Waist-to-height ratio	0.657 (0.612–0.703)	0.208	<0.001	0.355	-	0.001
Body mass index	0.618 (0.571–0.665)	0.007	0.001	0.513	0.001	-

AUC, area under the Receiver Operating Characteristic curve; BMI, body mass index; HC, hip circumference; WC, waist circumference; WHtR, waist-to-height ratio; WHR, waist-to-hip ratio, P-value for differences in the AUC.

### Optimal WC cut-off points to detect two or more non-adipose MS components

**Fig 2:**
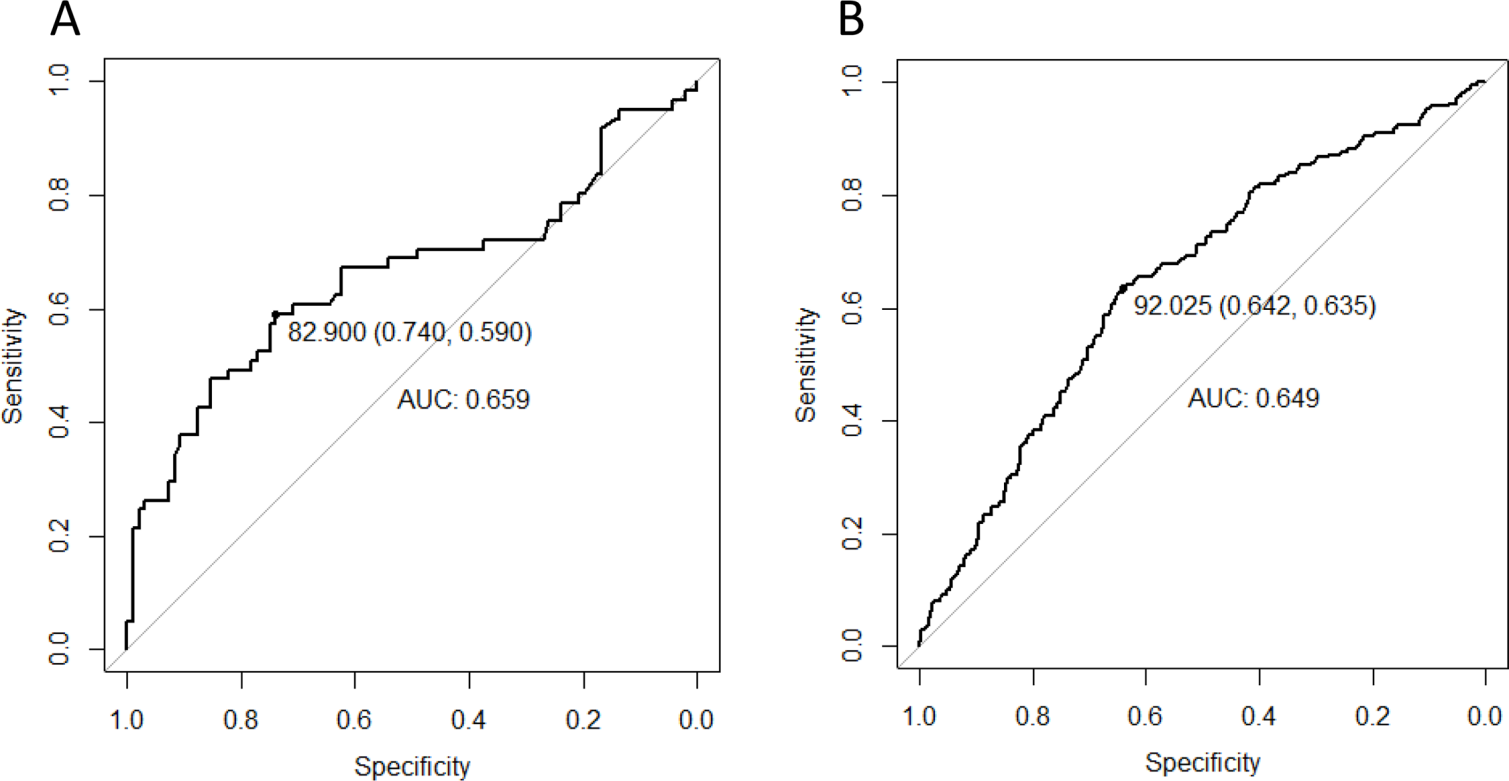
**Receiver operating characteristic (ROC) curve of the waist circumference in detecting the presence of two or more metabolic syndrome components in HIV-infected African men (A) and women (B).** The ROC curves show the optimal cutoff of 82.9 cm in men (sensitivity: 59%, specificity: 74%) corresponding to a c-statistic of 0.659 (A), and the optimal cutoff of 92.0 cm in women (sensitivity: 63.5%, specificity: 64.2%) corresponding to a c-statistic of 0.649 (B).

Fig 2 presents the results of the ROC analyses for WC in predicting the presence of ≥2 non-adipose MS components. The optimal cut-off points were 91.8 cm in the overall sample, 92 cm in women, and 83 cm in men using the closest-top-left point approach. The Youden’s index approach identified the same cut-off point in women, but a different cut-off point in men (87 cm). As showed in Table 3, the WC cut-off of 92 cm in women had the following performance: se 64%, sp 64%, Youden’s index 0.28, PPV 50%, NPV 76%, LR+ 1.77, LR- 0.57, DOR 3.12. In men, the WC cut-off of 83 cm had the following performance: se 59%, sp 74%, Youden’s index 0.33, PPV 59%, NPV 74%, LR+ 2.27, LR- 0.55, DOR 4.09; while equivalents for the cut-off point of 87 cm were: se 48%, sp 85%, Youden’s index 0.33, PPV 67%, NPV 72%, LR+ 3.26, LR- 0.61, DOR 5.31. When participants were stratified by duration on ART (at or above vs. below median duration), the optimal cut-off was mostly similar in men and women with longer vs. those with shorter duration on ART, and not appreciably different from the sex-specific thresholds obtained in the overall sample.

**Table 3:** Performance measures of different waist circumference cut-offs to predict two or more metabolic syndrome components in men and women.

Reference	Cut-off (cm)	Sensitivity (95%CI)	Specificity (95%CI)	PPV (95%CI)	NPV (95%CI)	LR+ (95%CI)	LR- (95%CI)	Diagnostic accuracy (95%CI)	DOR (95%CI)	Youden-index (95%CI)	NND (95%CI)
**Men**											
Mabchour [9]	80	0.62 (0.49–0.74)	0.64 (0.53–0.73)	0.52 (0.40–0.64)	0.73 (0.62–0.82)	1.71 (1.23–2.37)	0.59 (0.42–0.85)	0.63 (0.55–0.71)	2.88 (1.48–5.59)	0.26 (0.02–0.48)	3.87 (2.10–48.62)
Current study	82.9	0.59 (0.46–0.71)	0.74 (0.64–0.82)	0.59 (0.46–0.71)	0.74 (0.64–0.82)	2.27 (1.52–3.37)	0.55 (0.40–0.77)	0.68 (0.60–0.75)	4.09 (2.06–8.10)	0.33 (0.10–0.54)	3.03 (1.86–10.33)
Peer [8]	83.9	0.52 (0.39–0.65)	0.76 (0.66–0.84)	0.58 (0.44–0.71)	0.72 (0.62–0.80)	2.19 (1.43, 3.36)	0.63 (0.47–0.83)	0.67 (0.59–0.74)	3.50 (1.76–6.96)	0.29 (0.06–0.49)	3.51 (2.02–18.12)
Motala [6]	86	0.49 (0.36–0.62)	0.82 (0.73–0.89)	0.64 (0.49–0.77)	0.72 (0.62–0.80)	2.78 (1.68–4.58)	0.62 (0.47–0.80)	0.69 (0.62–0.77)	4.50 (2.18–9.29)	0.31 (0.09–0.52)	3.18 (1.94–10.74)
Current study	87	0.48 (0.35–0.61)	0.85 (0.77–0.92)	0.67 (0.51–0.81)	0.72 (0.63–0.80)	3.26 (1.88–5.66)	0.61 (0.48–0.79)	0.71 (0.63–0.78)	5.31 (2.49–11.32)	0.33 (0.11–0.53)	3.03 (1.90–8.82)
Prinsloo [16]; Kalk [17]; Matsha [7]	90	0.43 (0.30–0.56)	0.88 (0.79–0.93)	0.68 (0.51–0.82)	0.71 (0.62–0.79)	3.41 (1.86–6.24)	0.66 (0.52–0.82)	0.70 (0.62–0.77)	5.20 (2.36–11.45)	0.30 (0.09–0.49)	3.32 (2.03–10.84)
JIS 2009 [1]	94	0.30 (0.19–0.43)	0.92 (0.84–0.96)	0.69 (0.48–0.86)	0.67 (0.58–0.75)	3.54 (1.64–7.64)	0.77 (0.65–0.91)	0.68 (0.60–0.75)	4.60 (1.86–11.43)	0.21 (0.03–0.39)	4.72 (2.57–36.29)
**Women**											
JIS 2009 [1]	80	0.85 (0.80–0.90)	0.32 (0.27–0.37)	0.41 (0.36–0.46)	0.80 (0.72–0.86)	1.26 (1.15–1.37)	0.46 (0.32–0.65)	0.51 (0.47–0.55)	2.75 (1.77–4.25)	0.17 (0.07–0.27)	5.74 (3.72–13.82)
Matsha [7]	90	0.66 (0.59–0.72)	0.59 (0.54–0.64)	0.47 (0.41–0.53)	0.76 (0.70–0.80)	1.60 (1.37–1.87)	0.58 (0.47–0.71)	0.61 (0.57–0.65)	2.77 (1.95–3.94)	0.25 (0.13–0.36)	4.03 (2.76–7.77)
Crowther [5]	91.5	0.65 (0.58–0.71)	0.62 (0.57–0.67)	0.49 (0.43–0.55)	0.76 (0.71–0.81)	1.71 (1.46–2.02)	0.56 (0.46–0.69)	0.63 (0.59–0.67)	3.03 (2.14–4.31)	0.27 (0.15–0.38)	3.70 (2.61–6.62)
Current study; Motala [6]	92.05	0.64 (0.57–0.70)	0.64 (0.59–0.69)	0.50 (0.44–0.56)	0.76 (0.71–0.81)	1.77 (1.50–2.10)	0.57 (0.47–0.69)	0.64 (0.60–0.68)	3.12 (2.20–4.43)	0.28 (0.16–0.39)	3.61 (2.56–6.34)
Peer [8]	94	0.55 (0.48–0.62)	0.68 (0.63–0.73)	0.49 (0.42–0.55)	0.73 (0.68–0.78)	1.73 (1.43–2.09)	0.66 (0.56–0.78)	0.63 (0.59–0.67)	2.61 (1.85–3.70)	0.23 (0.11–0.35)	4.32 (2.89–8.92)
Prinsloo [16]	98	0.41 (0.34–0.48)	0.78 (0.73–0.82)	0.51 (0.43–0.58)	0.70 (0.66–0.75)	1.84 (1.44–2.37)	0.76 (0.67–0.86)	0.65 (0.61–0.68)	2.42 (1.68–3.49)	0.19 (0.07–0.30)	5.36 (3.37–13.43)

Sensitivity = TP/(TP+FN); Specificity = TN/(TN+FP); PPV, positive predictive value = TP/(TP+FP); NPV, negative predictive value = TN/(TN+FN); LR+, likelihood ratio positive = sensitivity/1-specificity; LR-, likelihood ratio negative = 1-sensitivity/specificity; Diagnostic accuracy = (TP+TN)/(TP+TN+FP+FN); DOR, diagnostic odds ratio = LR+/LR-; Youden’s index = (sensitivity + specificity)– 1; NND, number needed to diagnose = 1/Youden’s index, where TP, true positive; FP, false positive; TN, true negative; FN, false negative.

The performances of various cut-off points including the internationally advocated and those recommended by African studies for diagnosing MS are presented in Table 3. Compared with the derived cut-off points, the internationally advocated cut-off point of 94 cm in men showed higher specificity (92%) but lower sensitivity (30%), whereas in women the WC cut-off of 80 cm produced a lower specificity (32%), though a higher sensitivity (85%) in the current study sample. The Youden’s indices were 0.21 and 0.17 for the cut-off points of 94 cm and 80 cm in men and women, respectively.

The African-specific thresholds generated diagnostic performances close to those of our derived cut-off points, particularly for the cut-off values of 84 cm (Youden’s index 0.29, se 52%, sp 76%), 86 cm (Youden’s index 0.31, se 49%, sp 82%), and 90 cm (Youden’s index 0.30, se 43%, sp 88%) in men; and the cut-off points of 90 cm (Youden’s index 0.25, se 66%, sp 59%), 91.5 cm (Youden’s 0.27, se 65%, sp 62%), and 94 cm (Youden’s index 0.23, se 55%, sp 68%) in women; Table 3.

Based on the JIS criteria, the MS prevalence was 16.6% in men and 31.3% in women in our sample, Table 4. When applying the derived WC thresholds, the MS prevalence would increase to 24.2% (95% CI: 17.5–30.9) in men but decrease to 25.6% (22–29.1) in women. The prevalence of MS based on the other African-specific WC cut-off points are shown in Table 4.

**Table 4:** Comparison of the MS prevalence using the JIS waist circumference thresholds, and the derived cutoffs in this and other African populations.

Reference	Men		Women		p-value	Overall prevalence
	WC cutoff	prevalence	WC cutoff	prevalence		
JIS 2009*	94 cm	16.6 (10.8–22.4)	80 cm	31.3 (27.6–35.0)	<0.001	28.2 (25.0–31.4)
Modified JIS:						
Current study	83 cm	24.2 (17.5–30.9)	92 cm	25.6 (22–29.1)	0.730	25.3 (22.2–28.4)
Current study	87 cm	19.8 (13.5–26.0)	92 cm	25.6 (22–29.1)	0.132	24.3 (21.3–27.4)
Motala [6]	86 cm	20.4 (14.1–26.7)	92 cm	25.6 (22–29.1)	0.181	24.5 (21.4–27.6)
Matsha [14]	90 cm	18.5 (12.4–24.5)	90 cm	26.1 (22.5–29.6)	0.049	24.5 (21.4–27.6)
Peer [8]	84 cm	21.7 (15.2–28.1)	94 cm	22.8 (19.5–26.2)	0.752	22.6 (19.6–25.6)
Prinsloo [16]	90 cm	18.5 (12.4–24.5)	98 cm	19.0 (15.8–22.1)	0.891	18.9 (16.1–21.7)
El Mabchour [9]	80 cm	25.5 (18.7–32.3)	94 cm	22.8 (19.5–26.2)	0.488	23.4 (20.4–26.4)

*metabolic syndrome (MS) based on JIS criteria: ≥3 of waist circumference (WC) ≥94cm in men & ≥80cm in women, blood pressure ≥130/85mmHg or on hypertensive medication, HDL-C, high-density lipoprotein-cholesterol <1.03mmol/L in men & <1.3mmol/L in women, triglycerides ≥1.7mmol/L, FPG, fasting plasma glucose ≥5.6mmol/L or on anti-diabetic medication. Modified JIS criteria using the waist circumference cutoffs recommended from African studies to replace the JIS waist circumference cutoffs.

## Discussion

This study is among the first to determine the optimal WC thresholds for diagnosing the MS in an HIV-infected African population. The findings demonstrate that the optimal WC cut-off points in both men and women do not accord with the internationally recommended criteria for African populations. Notably, the optimal WC thresholds described in this study approximate those reported in other African studies conducted in the general population. In women, the optimal WC cut-off points in this and other local studies of 92 cm and 90–98 cm, respecitvely, were higher than the 80 cm advocated by the JIS criteria [5, 6, 8, 14, 16]. In contrast to the women in this study whose optimal WC threshold was higher than the JIS values, the optimal WC cut-off points of 83–87 cm in men were lower than the JIS recommended criteria of 94 cm. These findings were nevertheless in keeping with the 80–90 cm WC cut-off thresholds described in other South African studies [6, 8, 14, 16, 17]. The optimal WC thresholds of 80 cm and 94 cm in men and women, respectively, in Benin and Haiti also approximated this and other regional studies [9].

In this study, the Youden’s index and the closest-top-left point criteria identified the same cut-off point for WC in women but two different cut-off points in men. This discrepancy may be related to the small sample of 157 men in this study. The inconsistency of optimal cut-off points using the two ROC based approaches has been reported previously [18]. Perkins and Schisterman examined the situations when the closest-top-left point and the Youden’s index (J point) criteria agreed and disagreed with each other using the data of the placenta growth levels to classify women with preeclampsia [18]. They showed that when equal weight is given to sensitivity and specificity, the “closest-top-left point” and the Youden’s index methods identify the same cut-off point as “optimal” in certain situations and different cut-off points in others. They further demonstrated that when the two approaches give different values, the cut-point resulting in J-point was the only “optimal cut-point” with respect to minimising the overall misclassification rates. The authors therefore suggested that the Youden’s index approach should be used to find the optimal cut-off point [18].

The small variations among the African-specific thresholds could be attributed at least in part to differences in methodological approaches. For example, while Matsha et al., [7] Peer et al., [8] and Motala et al. [6] determined the optimal WC cut-off points for the presence of ≥2 MS components, [6–8] Crowther and Norris determined the optimal WC cut-off points for ≥3 out of 4 MS components, [5] Prinsloo et al. used raised BP, [16] Kalk et al. used insulin resistance (IR) and triglycerides-to-HDL-C ratio, [17] while Mabchour et al. used elevated BP, total cholesterol-to-HDL-C ratio and IR as the outcome [9].

The measures of diagnostic accuracy indicate that the JIS-advocated thresholds for WC did not perform well in these study participants. The advocated WC threshold of 80 cm for women yielded considerably low specificity, resulting in an overestimation of the MS prevalence by about 22%. The cut-off of 94 cm in men generated very low sensitivity, confirming that cut-off would underestimate the prevalence of MS among HIV-infected African men. Indeed the prevalence of MS in men increased by nearly 20% when our Youden index based cut-off was applied, instead of the JIS one. The combination of the under-diagnosis in men and over-diagnosis in women using the internationally advocated thresholds, led to 16% overestimation of MS prevalence at the overall sample level, relative to estimates using the derived optimal cut-off points. Therefore, in addition to improving the gender specific allocation of interventions to mitigate diseases risk associated with the MS, applying the African specific cut-off points will likely be cost-saving as fewer people will require such interventions.

Results of our validation studies demonstrate that thresholds of 86–90 cm in men and 90–94 cm in women, derived from other African studies, performed well in this HIV-infected sample. The overall accuracies were close to that of the derived cut-off points shown in this study and better than those internationally advocated. Other investigators have suggested the unique WC threshold of 90 cm in men and women as a practical recommendation for MS screening in the general population in Africa [14]. Our validation studies would tend to support the application of those recommendations in people with HIV infection.

With regards to the use of specific measures of adiposity, the ROC curves showed that WC, WHR, WHtR and BMI were equally effective in men; while in women WC, along with WHR and WHtR, had a better ability to discriminate individuals with and without the MS compared to BMI. This is unsurprising since BMI measures total fat mass while WC assessed subcutaneous and intra-abdominal fat mass or visceral obesity, a key player in the aetiological pathways of the MS [3, 4]. Therefore, the findings suggest that WC is not only a simpler but also an accurate index to identify MS in HIV-infected individuals. Our findings are supported by those of Beraldo et al. who found that WC was a good tool for identifying individual cardio-metabolic risk factors as well as the MS in both genders in 280 Brazilians on ART [19].

### Strength and limitations

This is the first study attempting to investigate the relevance of WC and the applications of the recommended cut-off points including those internationally advocated and African-specific to predict the presence of MS in Africans living with HIV infection. Including participants from many primary health care facilities in both urban and rural areas is a major strength and will likely enhance the external validity of our findings. The small sample size, particularly in men, and the lack of external validation on another sample of people with HIV infection are the main limitations. Furthermore, in both HIV-infected people and the general population in Africa, follow-up studies are needed to determine the effect of baseline MS status on the incidence of major health outcomes reported elsewhere to be associated with MS, and to what extent those effects can be mitigated by lifestyle and pharmacological interventions.

## Conclusions

This study underlines the sub-optimal applicability of the currently advocated WC thresholds for MS diagnosis in HIV-infected African men and women, supporting the need to revisit the WC thresholds in use in African people including those living with HIV infection. Our study findings extend to African people with HIV infection, and together with reports from previous studies conducted in the general population across Africa, suggest that the uncritical application of the internationally advocated WC thresholds will lead to MS over-diagnosis in women and under-diagnosis in men. This will result in an overall over-diagnosis at the population level, and accordingly an overuse of health resources. Our study also provides additional support for the unique WC threshold of 90 cm in both men and women, as a practical and more accurate approach to MS diagnosis in African populations, including those living with HIV infection.
